# Butyric Acid Precursor Tributyrin Modulates Hippocampal Synaptic Plasticity and Prevents Spatial Memory Deficits: Role of PPARγ and AMPK

**DOI:** 10.1093/ijnp/pyac015

**Published:** 2022-02-13

**Authors:** Ana Belén Sanz-Martos, Jesús Fernández-Felipe, Beatriz Merino, Victoria Cano, Mariano Ruiz-Gayo, Nuria Del Olmo

**Affiliations:** Department of Health and Pharmaceutical Sciences, School of Pharmacy, Universidad CEU-San Pablo, CEU Universities, Madrid, Spain; Department of Health and Pharmaceutical Sciences, School of Pharmacy, Universidad CEU-San Pablo, CEU Universities, Madrid, Spain; Department of Health and Pharmaceutical Sciences, School of Pharmacy, Universidad CEU-San Pablo, CEU Universities, Madrid, Spain; Department of Health and Pharmaceutical Sciences, School of Pharmacy, Universidad CEU-San Pablo, CEU Universities, Madrid, Spain; Department of Health and Pharmaceutical Sciences, School of Pharmacy, Universidad CEU-San Pablo, CEU Universities, Madrid, Spain; Department of Health and Pharmaceutical Sciences, School of Pharmacy, Universidad CEU-San Pablo, CEU Universities, Madrid, Spain; Department of Psychobiology, School of Psychology, National Distance Education University (UNED), Madrid, Spain

**Keywords:** Tributyrin, spatial memory, LTP, hippocampus, synaptic transmission

## Abstract

**Background:**

Short chain fatty acids (SCFA), such as butyric acid (BA), derived from the intestinal fermentation of dietary fiber and contained in dairy products, are gaining interest in relation to their possible beneficial effects on neuropsychological disorders

**Methods:**

C57BL/6J male mice were used to investigate the effect of tributyrin (TB), a prodrug of BA, on hippocampus (HIP)-dependent spatial memory, HIP synaptic transmission and plasticity mechanisms, and the expression of genes and proteins relevant to HIP glutamatergic transmission.

**Results:**

Ex vivo studies, carried out in HIP slices, revealed that TB can transform early-LTP into late-LTP (l-LTP) and to rescue LTP-inhibition induced by scopolamine. The facilitation of l-LTP induced by TB was blocked both by GW9662 (a PPARγ antagonist) and C-Compound (an AMPK inhibitor), suggesting the involvement of both PPARγ and AMPK on TB effects. Moreover, 48-hour intake of a diet containing 1% TB prevented, in adolescent but not in adult mice, scopolamine-induced impairment of HIP-dependent spatial memory. In the adolescent HIP, TB upregulated gene expression levels of *Pparg*, leptin, and adiponectin receptors, and that of the glutamate receptor subunits AMPA-2, NMDA-1, NMDA-2A, and NMDA-2B.

**Conclusions:**

Our study shows that TB has a positive influence on LTP and HIP-dependent spatial memory, which suggests that BA may have beneficial effects on memory.

**Significance Statement**

The current study investigated the effect of tributyrin, a prodrug of butyric acid, on memory processes. We found that tributyrin elicited favorable changes both in memory and hippocampal long-term potentiation, which suggests that butyric acid, derived from dietary fiber and contained in dairy products, may be endowed with therapeutic potential for the management of memory impairment. As far as we know, our study is a first attempt to investigate the effect of oral tributyrin on mechanisms underlying cognitive processes and the mechanism of action of this drug in the modulation of hippocampal plasticity.

## Introduction

Short-chain fatty acids (SCFA) are fatty acids with fewer than 6 carbon atoms, which are contained in dairy products and generated as end metabolites of dietary fiber fermentation by gut microbiota (Morrison and Preston, 2016). SCFA promote satiety and have a complex effect on adipogenesis since they participate in adipocyte differentiation, triglyceride synthesis, adipose tissue angiogenesis, and energy metabolism (Canfora et al., 2015). Their actions are mediated by FFAR2, FFAR3, and GPR109A receptors (Priyadarshini et al., 2018). Moreover, SCFA have been shown to improve insulin resistance and systemic inflammation associated with obesity in humans (Chambers et al., 2019).

Related with that, butyric acid/butyrate (BA), an SCFA particularly abundant in cow milk fat and therefore an integral part of the western diet, is an inhibitor of histone deacetylase (Davie, 2003; Szentirmai et al., 2019), like other SCFA, which have been shown to induce neuronal differentiation and neuroprotection (Shukla and Tekwani, 2020). Accordingly, SCFA have been proposed as a potential tool to mitigate memory impairment related to neurodegenerative diseases, such as Alzheimer’s disease (AD) (Govindarajan et al., 2011; Lei et al., 2016). Specifically, BA enhances learning and memory in mice that underwent traumatic brain injury (Dash et al., 2009; Lei, Vacy and Boon, 2016).

The hippocampus (HIP) seems to be an important target for BA, as suggested by studies showing that BA increases granular cell layer volume and neurogenesis in this brain area (Val-Laillet et al., 2018). Moreover, BA improves memory in rats submitted to sepsis (Steckert et al., 2015), prevents memory decline in aged rats (Garcez et al., 2018), and enhances the formation of long-term memory in young rats (Levenson et al., 2004). The mechanisms underlying these effects have not been identified, but the current knowledge on this topic points to the involvement of PPARγ (Mishra et al., 2014). BA intake, combined with the PPARγ agonist pioglitazone, has been shown to protect against HIP neuroinflammation and neuronal loss in animals with cognitive impairment induced by a high-fructose diet (Li et al., 2019). This finding is coherent with the positive effect that PPARγ activation has on spatial cognitive deficits and LTP impairment (Zhou et al., 2016) as well as with the attenuation of LTP decline triggered by βA peptide within the HIP (Costello et al., 2005).

It is noteworthy that PPARγ agonists modulate the activity of AMPK (Lee and Kim, 2010), a Ser/Thr kinase pivotal for energy homeostasis (Hardie et al., 2012), which also plays a critical role in consolidating long-lasting synaptic plasticity and long-term memory (Costa-Mattioli et al., 2009). AMPK activation ameliorates spatial memory deficits in Alzheimer’s disease models (Du et al., 2015). Moreover, AMPK has been shown to be essential to maintain neuronal energy levels during synaptic activation and, consistently, its inhibition by C-Compound (C-C) impairs HIP synaptic plasticity and long-term memory formation (Marinangeli et al., 2018).

The aim of the current study has been to characterize the effect of tributyrin (TB), a prodrug of BA (Su et al., 2004), on HIP-dependent spatial memory, HIP synaptic plasticity, and expression of genes and proteins related with synaptic transmission. TB is endowed with less toxicity and better pharmacokinetic properties than its active metabolite (Egorin et al., 1999). Moreover, BA has been shown to induce avoidance behavior and anxiogenic effects because of its unpleasant flavor (Wallace and Rosen, 2000; Hebb et al., 2002), so its oral administration may have an impact on food intake. Since lipoprotein lipase has been identified in the blood-brain barrier, and particularly in the HIP (Picard et al., 2014), one can assume that oral TB supplies the brain with BA.

## METHODS

### Animals and Experimental Design

**Figure 1.**
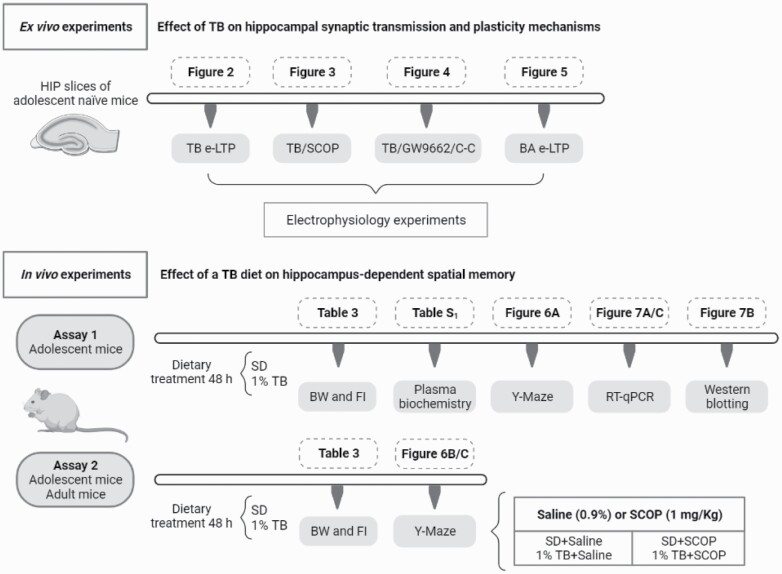
Experimental design. Ex vivo electrophysiological experiments, aimed at characterizing the effect of tributyrin (TB) on HIP (hippocampal) synaptic transmission and plasticity mechanisms, were conducted in HIP slices from adolescent mice. Moreover, we have carried out in vivo experiments to identify the effect of a TB diet on HIP-dependent spatial memory and on the expression of genes and proteins relevant to HIP glutamatergic transmission, and other receptors, such as PPARγ.

C57BL/6J male mice (Charles River, Châtillon, France) were housed in individual cages under a 12-hour-light/-dark cycle in a temperature-controlled room (22ºC) with food and water available ad libitum. The investigation conformed to the Guide for the Care and Use of Laboratory Animals published by European Union Directive (2010/63/EU) and was approved by the Committee on Animal Research and Ethics of the San Pablo-CEU University (PCD-CEU08-112-16). The experimental design is illustrated in Figure 1 and consisted of ex vivo and in vivo assays.

Ex vivo studies were performed in HIP slices obtained from 5-week-old naïve mice that were incubated with either TB or BA to analyze the effect of these drugs on HIP synaptic transmission and plasticity mechanisms. Additionally, the effect of TB on scopolamine (SCOP)-induced LTP impairment was also investigated. The involvement of PPARγ and AMPK on TB effects was studied by using GW9662 (a PPARγ antagonist) and C-C (an AMPK inhibitor), respectively. Stock solutions of SCOP hydrobromide, TB, and BA were prepared in Krebs–Ringer bicarbonate (KRB) buffer, GW9662, and C-C solutions in dimethyl sulfoxide (DMSO; <0.05% in all cases). DMSO (0.05%) was added in the corresponding control experiments. All drugs were added to the perfusion chamber immediately after baseline period, and high-frequency stimulation (HFS) was applied 20 minutes after starting drug perfusion. In all cases, 20 minutes after HFS, the drug was removed and the slice washed with KRB. Drugs were purchased from Sigma (Saint Louis, CO, USA).

In vivo studies, which were carried out in 5-week-old mice that had consumed (48 hours) standard chow containing 1% TB, aimed at identifying the effect of TB on HIP-dependent spatial memory (Y maze test) as well as on the expression of genes and proteins relevant to HIP glutamatergic transmission. PPARγ expression was also quantified. Briefly, after spatial memory assessment, animals were killed, blood collected, and HIP and prefrontal cortex (PFC) dissected and frozen until gene and protein expression determination (Assay 1). A second assay was performed in both adolescent (5 weeks old) and adult mice (11 weeks old) to characterize the effect of a diet containing 1% TB on SCOP-induced memory impairment. In this case, mice were administered (i.p.) either with saline or SCOP, and, after 30 minutes, spatial memory was assessed in the Y maze (Assay 2; Figure 1). In both studies, animals were randomly assigned (48 hours, free access) either to standard chow (SD, Teklad Global 2018, Harlan Laboratories) or to a diet containing 1% TB. The dose of TB was selected in accordance with previous studies in rats receiving 1 g/kg of oral TB (Egorin et al., 1999; Miyoshi et al., 2015, 2020). Under our conditions, the estimated daily consumption of TB was 0.035 g, which corresponds approximately to 1.8 g/kg of TB consumed within 48 hours. Body weight (BW; expressed in grams) and food intake (FI; expressed in kilocalories) were monitored (Table 3). The TB-enriched diet was manufactured in our animal facility with standard chow (99%; 3.1 kcal/g) and TB (1%; 7 kcal/g).

### Electrophysiological Experiments

The ex vivo effect of TB on HIP synaptic transmission and plasticity was studied in HIP slices obtained from naïve mice (a single slice from each individual animal was considered as n = 1). Transverse HIP slices (400 µm) were prepared using a manual tissue chopper (Stoelting Tissue Slicer, IL) and placed in gassed (95% O_2_, 5% CO_2_) ice-cold KRB containing (mM) 109 NaCl, 2.5 KCl, 1 KH_2_PO_4_, 1.3 MgSO_4_, 2.5 CaCl_2_, 26.2 NaHCO_3_, and 11 glucose in a humidified interface chamber at 20ºC –25ºC, as described previously (Del Olmo et al., 2000; Valladolid-Acebes et al., 2013). After 3-hour incubation, slices were transferred to a submersion recording chamber that was perfused with KRB at a rate of 1.8–2 mL/min. Field excitatory postsynaptic potentials (fEPSPs) were evoked by stimulating Schaffer collateral-commissural fibers with biphasic electrical pulses (30–70 μA, 100 μs, 0.033 Hz) delivered through bipolar tungsten insulated microelectrodes (0.5 MΩ), and they were recorded in the CA1 stratum radiatum using tungsten electrodes (1 MΩ). Electrical pulses were generated by a pulse generator Master 8 (AMPI, Israel), and the recording electrode was connected to an AI-402 amplifier (Axon Instruments, USA) connected in turn to a CyberAmp 320 signal conditioner (Axon Instruments). Stimulus intensity was adjusted to induce a 30%–40% maximal fEPSP slope. LTP was induced by 1 (to obtain an early-LTP that did not last >90 minutes in normal conditions) or 4 (to get a late-LTP, which maintains >180 minutes in standard conditions) HFS trains (100 Hz, 1 second) respectively, separated by 20 seconds. Synaptic strength was assessed by measuring the initial slope of the fEPSP using pCLAMP 9.0 software, and data were normalized with respect to the mean values of the responses of each animal during the 20-minute baseline period. All the assays were carried out at 32°C. Relationships between fEPSP slope and stimulation strength (input/output curves [I/O]; 0–100 μA) were applied to evaluate basal synaptic transmission (BST).

### Spontaneous Alternation in the Y Maze

The effect of TB on spatial memory was characterized by using the Y maze test. Spatial working memory was analyzed by recording spontaneous alternation in a black plexiglass Y maze consisting of 3 identical arms (50 cm long × 19 cm wide) with 35-cm-high walls (Contreras et al., 2019). Animals were placed in the testing room, equipped with visual cues, 60 minutes before testing for habituation. For testing, mice explored the maze for 10 minutes, during which arm entries (4 paws within an arm) were recorded. A correct alternation was defined as an entry into 3 different arms (A, B, and C) in overlapping successive sequences of 3 arm entries. The percent alternation score was calculated as [(actual alternations)/(possible alternations)] × 100.

A first study was carried out in 5-week-old mice that consumed either SD (n = 6) or 1% TB diet (n = 7) over 48 hours (Assay 1). A second study was performed in adolescent (5 weeks old; n = 32) and adult mice (11 weeks old; n = 32) adhered to the same dietary treatment and subjected to SCOP treatment (1 mg/kg), based on (Bak et al., 2017), according to the following schedule: SD diet + saline (n = 8), SD diet + SCOP (n = 8), 1% TB diet + saline (n = 8), 1% TB diet + SCOP (n = 8). Spatial memory was assessed 30 minutes after SCOP/vehicle administration (Assay 2).

### Real-Time Reverse Transcription Polymerase Chain Reaction (RT-qPCR)

Total RNA was extracted from whole PFC and HIP samples (Assay 1) by using the Tri-Reagent protocol (Life Technologies, Barcelona, Spain). cDNA was then synthesized from 1 µg total mRNA by using a cDNA RT kit (Bio-Rad, Barcelona, Spain). RT-qPCR was performed by using designed primer pairs (Integrated DNA Technologies, USA; Table 1). SsoAdvanced Universal SYBR Green Supermix (Bio-Rad, Madrid, Spain) was used for amplification according to the manufacturer’s protocols in an ABI PRISM 7000 Sequence Detection System (Applied Biosystems, MA, USA). Gene values were normalized to the reference genes *18s* and *β-Actin*. The 2^-∆∆*C*t^ method was used to determine relative expression levels. Statistics were performed using 2^-∆∆*C*t^ values (Schmittgen and Livak, 2008).

**Table 1.** Designed Primer Pairs Used in This Study

Genes	Forward	Reverse
*18-S*	GGGAGCCTGAGAAACGGC	GGGTCGGGAGTGGGTAATTT
*Actb*	TGGTGGGAATGGGTCAGAAGGACTC	CATGGCTGGGGTGTTGAAGGTCTCA
*Gria1*	CAGGTGCGTTTTGAAGGTTTGACAG	CGTATTTGCCGTCGCTGACAATCTC
*Gria2*	ACAGTGCATTTCGGGTAGGG	CCTTTGAGGTCAGGTCGCAT
*Grin1*	GACTGGCCGTGTGGAATTCAATGAG	CACTATCTTTAGTCTGGTGGACATCTG
*Grin2A*	GAGCGTTCAGAAGTGGTGGA	ACGAAGACAGCAATGGCAGA
*Grin2B*	AGGTCTTTGCTTCTACGGGC	GCTGGCTGCTCATAACCTCA
*Slc1a3*	GCAAGACACTGACACGCAAG	ATACGGTCGGAGGGCAAATC
*Ppara*	GTACGGCAATGGCTTTATCACACGC	GAAGGTGTCATCTGGATGGTTGCTC
*Pparg*	CATGGTTGACACAGAGATGCCATTCTG	TTGATCGCACTTTGGTATTCTTGGAGC
*Lepr*	GGCACCATTTCCGCTTCAAT	TCTCTTGCTCCTCACCTGGA
*Adipor1*	CCTGGCTCTATTACTCCTTC	GAACACTCCTGCTCTTGTCT
*Adipor2*	ACTCTGGTCTACAACTCTGACA	GTGTTTGGCTGGCTCGTTC
*Ffar3*	ACCTGACCATTTCGGACCTG	TGAAGGGCAGAAGCCATCTC

### Western-Blot Assays

Whole HIP samples (Assay 1) were homogenized in ice-cold lysis buffer (in mM; 20 Tris-HCl pH 7.4, 100 KCl, 5 NaCl, 2 EDTA, 1 EGTA, 250 sucrose, 2 dithiothreitol, 2 phenylmethylsulphonyl fluoride) containing 1 mg/mL aprotinin, 1 mg/mL leupeptin, and 50 mg/mL N^α^-tosyl-L-lysine chloromethyl ketone hydrochloride. Protein concentration was measured using the Bradford method (Bradford, 1976). Equal amounts of protein (30 µg) were mixed with Laemmli buffer, then loaded on an sodium dodecyl sulfate–polyacrylamide gel electrophoresis gel and subjected to electrophoresis. Proteins were transferred to nitrocellulose membranes (GE Healthcare, Barcelona, Spain) by using a transblot apparatus (Bio-Rad, Madrid, Spain). Membranes were blocked with skimmed milk powder (5%) in Tween-PBS for 1 hour. Primary antibodies against NMDAR1 (1:200), NMDAR2A (1:2000), NMDAR2B (1:500), AMPAR1 (1:1000), and AMPAR2 (1:500) were applied overnight (4°C) (Table 2). After washing, appropriate secondary antibodies (anti-rabbit/mouse/goat IgG-peroxidase conjugated; 1:5000) were added for 1 hour (Table 2). Blots were incubated in enhanced chemiluminescence reagent (ECL Prime; GE Healthcare) and developed using the ChemiDoc XRS+ Imaging System (BioRad, Madrid, Spain). To check the equal loading of samples, blots were re-incubated with β-actin antibody.

**Table 2.** Antibodies Used in the Study

Antibody	Type	Reference
Anti AMPAR1	Mouse monoclonal	MAB2263 (Merck Millipore)
Anti AMPAR2	Rabbit monoclonal	49694 (Signalway Antibody)
Anti NMDAR1	Rabbit monoclonal	ab68144 (Abcam)
Anti NMDAR2A	Rabbit monoclonal	ab124913 (Abcam)
Anti NMDAR2B	Mouse monoclonal	ab28373 (Abcam)
β-actin	Mouse monoclonal	A5316 (Sigma)
Anti-mouse IgG	Goat monoclonal	SC-516102 (Santa Cruz Biotechnology)
Anti-rabbit IgG	Mouse monoclonal	SC-2357 (Santa Cruz Biotechnology)

### Plasma Biochemistry

Glucose (Biolabo, Maizy, France), triglycerides (Spinreact, Madrid, Spain), and non-esterified free fatty acids (Wako Bioproducts, Virginia, USA) were measured by colorimetric methods. Leptin, insulin, and adiponectin levels were analyzed by EIA (Biovendor, Brno, Czech Republic, Mercodia, Uppsala, Sweden and Abcam, Cambridge UK, respectively) (supplementary Table 1). These analyses were carried out in plasma samples from animals of the first assay.

### Statistical Analysis

Two-way ANOVA was used to detect differences in the FI and Y maze test (Assay 2). Statistical differences of individual effects of BW, plasma parameters, Y maze test (Assay 1), and gene/protein expression levels were evaluated by 2-tailed Student’s *t* test. Electrophysiological results were assessed by 1- or 2-way ANOVA, and 2-tailed Student’s *t* test were used when indicated. Data are presented as mean ± SEM (post-hoc Bonferroni test correction). *P < *.05 was considered statistically significant. In bar graphs of electrophysiological results, potentiation level is expressed as the mean of 10 consecutive minutes. Outliers were identified by using the ROUT method (Q = 1%). Statistics were performed using GraphPad Prism 7.0 software (Inc. USA).

## RESULTS

### TB Potentiated Synaptic Transmission Within the Hippocampus and Transformed e-LTP Into l-LTP

**Figure 2.**
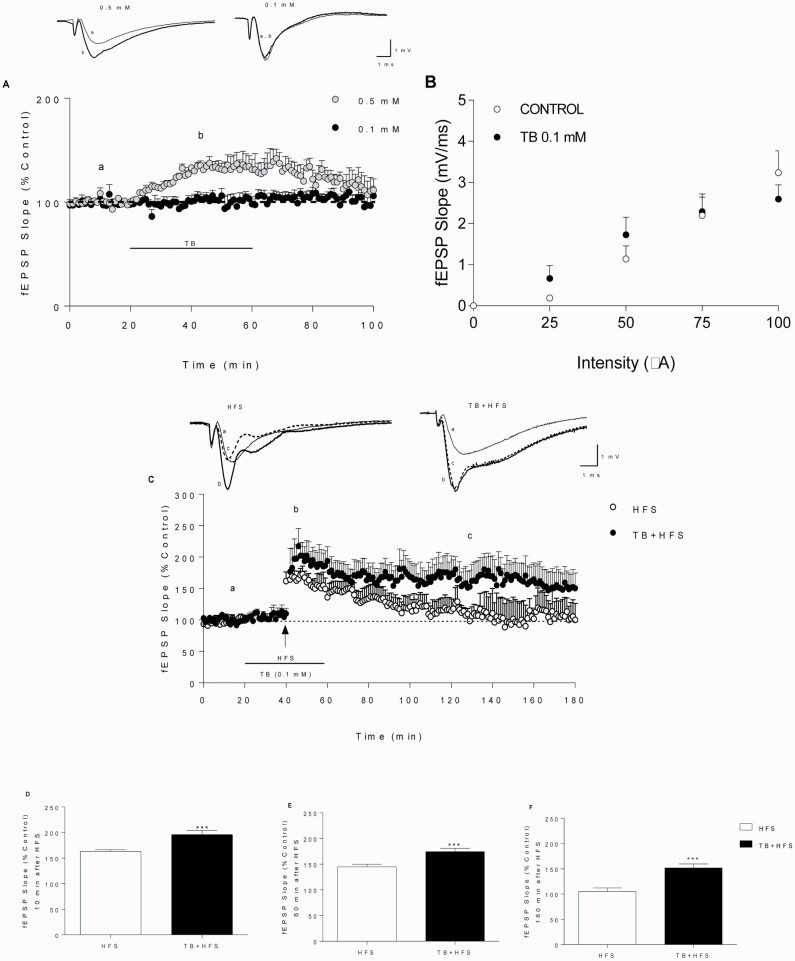
TB converts early-long term potentiation (e-LTP) to late long term potentiation (l-LTP) (A) Time-course of field extracellular postsinaptic potential (fEPSP) slope during/after perfusion with 0.5 mM (n = 3, gray circles) and 0.1 mM TB (n = 6, black circles). (B) Plots correspond to I/O curves in control (white circles, n = 10) and TB-perfused slices (black circles, n = 5). (C) Time-course of fEPSP slope changes induced by a single HFS (100 Hz, 1 second, black arrow) in controls (high frequency stimulation [HFS], n = 9, white circles) and samples perfused with 0.1 mM TB (n = 6, black circles). The horizontal line represents the duration of TB perfusion. (A, C) The upper traces correspond to fEPSPs recorded during the basal period (a, thin trace), following HFS (b, thick trace) and final recording (c, dash trace) of a representative experiment for each case. Calibration: 1 mV, 1 ms. (D) Comparison of fEPSP slope potentiation (mean ± SEM of 10 consecutive minutes) considered post-tetanic potentiation (PTP) (E) 60 minutes after HFS and (F) 160 minutes after HFS. Data are expressed as the mean ± SEM ***P < .001 compared with control group (2-tailed Student’s *t* test, 2-way ANOVA followed by post-hoc Bonferroni’s test).

Extracellular recordings were conducted to study the ex vivo effect of TB (0.1 and 0.5 mM) on synaptic transmission and plasticity in the HIP CA1 area. In all the studies, TB was used at 0.1 mM since this concentration did not modify the time-course of the fEPSP slope (Figure 2A) or BST (Figure 2B). In contrast, a higher concentration of TB (0.5 mM) potentiated the fEPSP slope, leading to a short-term LTP-like phenomenon (Figure 2A). On this basis, 0.1 mM TB was used in all the following experiments. As appears illustrated in Figure 2C, e-LTP, triggered by a single HFS pulse, turned towards a l-LTP in the presence of 0.1 mM TB. TB-induced potentiation of fEPSP was detected 10 minutes after HFS application, which corresponds to post-tetanus potentiation (PTP) (Figure 2D; control vs TB; *t*_(148)_ = 3.948, *P < *.05), and was maintained during 60 (Figure 2E; control vs TB; *t*_(148)_ = 3.786, *P < *.001) and 160 additional minutes (Figure 2F; control vs TB; *t*_(88)_ = 3.964, *P < *.001).

### TB Rescued SCOP-Induced Impairment of Hippocampal LTP

**Figure 3.**
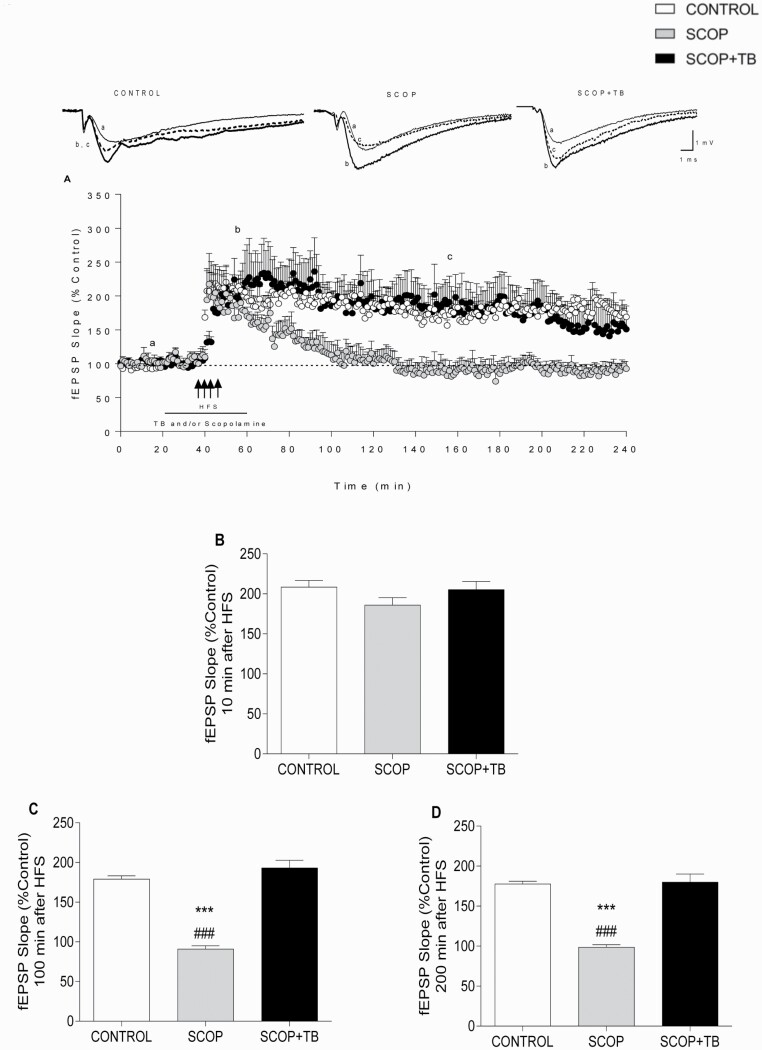
Effect of TB on scopolamine (SCOP)-induced impairment of LTP in HIP CA1 slices. (A) Effect of four 100-Hz pulses separated by 20 seconds (indicated by 4 arrows) on fEPSP slope in control (white circles, n = 6) and slices perfused with either 0.1 mM SCOP (gray circles, n = 5) or 0.1 mM TB + 0.1 mM SCOP (black circles, n = 6). The horizontal line represents the time during which TB and/or SCOP were perfused. The upper traces correspond to fEPSPs recorded during the basal period (a, thin trace), following HFS (b, thick trace), and final recording (c, dash trace) of a representative experiment for each case. Calibration: 1 mV, 1 ms. Comparison of fEPSP potentiation level (B) 10 minutes, (C) 100 minutes, and (D) 200 minutes after HFS. Data are expressed as means ± SEM. ***P < .001 compared with control group; ^###^*P < *.001 compared with SCOP+TB group (1-way or 2-way ANOVA followed by post-hoc Bonferroni’s test).

Since SCOP is a potent inhibitor of HIP LTP (Calabresi et al., 1999; Sánchez et al., 2009; Portero-Tresserra et al., 2014), we wanted to investigate the effect of TB on SCOP-induced impairment of LTP (Figure 3A). As detailed in Figure 3, 0.1 mM SCOP did not modify the baseline (Figure 3A). Moreover, SCOP did not modify l-LTP induction (Figure 3A–B) but fully abolished LTP maintenance (Figure 3A) measured 100 (Figure 3C) and 200 minutes after HFS application (Figure 3D). The impairment of LTP by SCOP was fully retrieved by 0.1 mM TB (Figure 3C–D). Figure 3B–D show the potentiation level of the fEPSP (% control) during 10 consecutive minutes measured 10 minutes (PTP; Figure 3B), 100 minutes (Figure 3C) and 200 minutes (Figure 3D) after HFS application (Figure 3C) (1-ANOVA F_(2,167)_ = 63.42, *P < *.001); Figure 3D; (1-ANOVA F_(2,167)_ = 46.02, *P < *.001). Curves I/O were carried out before HFS in all the experiments, and no statistical differences were found among the groups, showing that SCOP did not modify BST in the presence or absence of TB (2-ANOVA F_(8,10)_ = 0.2347, *P* = .9744).

### Effect of TB Involved PPARγ and Depended on AMPK Activation

**Figure 4.**
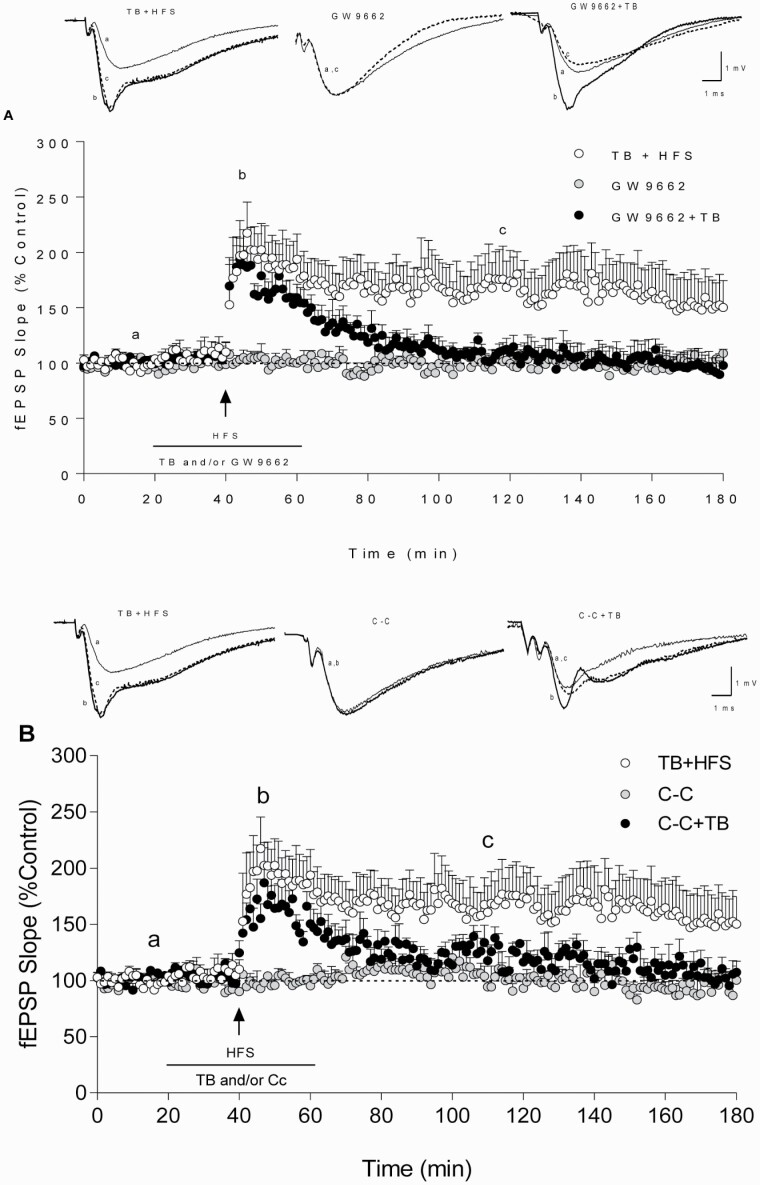
Both GW9662 and Compound-C inhibit TB-induced LTP facilitation. Effect of a single 100-Hz pulse (e-LTP, 1 arrow) on the fEPSP slope in HIP CA1 slices perfused with 0.1 mM TB (white circles, n = 6, data from Figure 2C) and either 0.1 mM TB + 2 μM GW9662 (black circles, n = 5) (A) or 0.1 mM TB **+** 5 μM, C-C (black circles, n = 5) (B). Gray circles correspond to fEPSP slopes in GW9662 (n = 3; A) and C-C (n = 3, B) treated slices without HFS application. The horizontal line represents the duration of TB and/or GW9662/C-C perfusion. The upper traces are representative fEPSPs, recorded during the basal period (a, thin trace), following HFS (b, thick trace), and final record (c, dash trace) for each group. Calibration: 1 mV, 1 ms (A and B). Data are expressed as means ± SEM (2-way ANOVA followed by post-hoc Bonferroni’s test).

Extracellular recordings were conducted to study the effect of the PPARγ antagonist GW9662 (2 μM) on synaptic transmission and plasticity changes evoked by TB (0.1 mM). As shown in Figure 4A, GW9662 inhibited l-LTP facilitation evoked by TB (2-ANOVA TB+HFS vs GW9662+TB effect, F_(1,1437)_ = 363.9, *P < *.001). Data for TB+HFS experiments are obtained from data represented in Figure 2C. In control experiments for GW9662 (without HFS), any change in synaptic transmission was produced (Figure 4A).

Because LTP is an energy-demanding process and PPARγ agonists have been shown to modulate the activity of AMPK (Lee and Kim, 2010), we wanted to explore the involvement of this ubiquitous energy sensor on TB effects. With this aim, the effect of the AMPK inhibitor C-C (5 μM) was tested. As illustrated in Figure 4B, C-C blocked the effect of TB on l-LTP induction (2-ANOVA TB+HFS vs CC+TB effect, F_(1,1440)_ = 363.9, *P < *.001; comparison was made from the moment at which drug perfusion was completed until the end of recordings). Data for TB+HFS experiments were obtained from data represented in Figure 2C. Moreover, we carried out I/O curves in all the experiments, and neither GW9662 nor C-C modified BST in the presence or absence of TB (GW9662; 2-ANOVA F_(3,3)_ = 0.1617, *P* = .9156 and C-C; 2-ANOVA F_(4,2)_ = 0.6844, *P* = .6661).

Moreover, to assess whether l-LTP facilitation was NMDA-mediated, extracellular recordings were conducted to study the effect of the selective competitive NMDA receptor antagonist L-2-amino-5-phosphonovaleric acid (50 µM) on synaptic transmission and plasticity changes evoked by TB (0.1 mM). Results revealed that NMDA receptors are required for the TB-mediated conversion of e-LTP to l-LTP (data not shown).

### BA Produced the Same Effect on Synaptic Plasticity as TB

**Figure 5.**
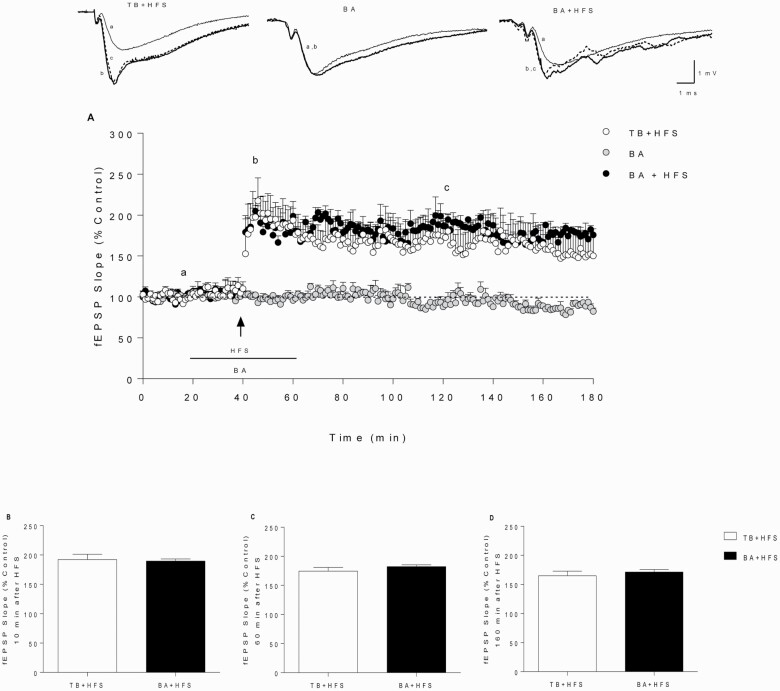
Effect of butyric acid on synaptic plasticity in HIP CA1 slices. The plots represent the time course of fEPSP changes induced by a single 100-Hz HFS train (1 second) (e-LTP, black arrow) in control slices. BA (75 μM, gray circles, n = 4) and BA (75 μM) one 100-Hz pulse (black circles, n = 5) compared with one 100-Hz pulse in the presence of TB 0.1 mM (white circles, n = 6, data from Figure 2C). Horizontal lines represent the duration of BA or TB perfusion. The upper traces are averages of the fEPSPs recorded during the basal period (a, thin trace), following HFS (b, thick trace), and final recording (c, dash trace). Calibration: 1 mV, 1 ms (A). Comparison of fEPSP slope potentiation 10 minutes (PTP) (B), 60 minutes after HFS (C), and 160 minutes after HFS (D). Data are expressed as the means ± SEM (2-tailed Student’s *t* test, 2-way ANOVA followed by post-hoc Bonferroni’s test).

Since TB is a prodrug of BA (Su et al., 2004), a control assay to study the direct effect of BA on synaptic plasticity was carried out. As shown in Figure 5, BA (75 μM) failed to alter the fEPSP slope (Figure 5A) but, similarly to TB (Figure 2C), turned toward l-LTP the e-LTP triggered by a single HFS pulse (Figure 5A). The application of a single HFS train in the presence of BA provoked the same effect as TB (Figure 2C; no statistical differences) on both PTP (Figure 5B) and fEPSP maintenance (Figure 5C–D). Curve I/O was carried out during BA application, and no statistical differences were found in BST between BA application and TB (0.1 mM) (2-ANOVA F_(3,8)_ = 2.012, *P* = .1909).

### TB Reversed SCOP-Induced Memory Impairment in Adolescent Mice in the Y Maze

**Figure 6.**
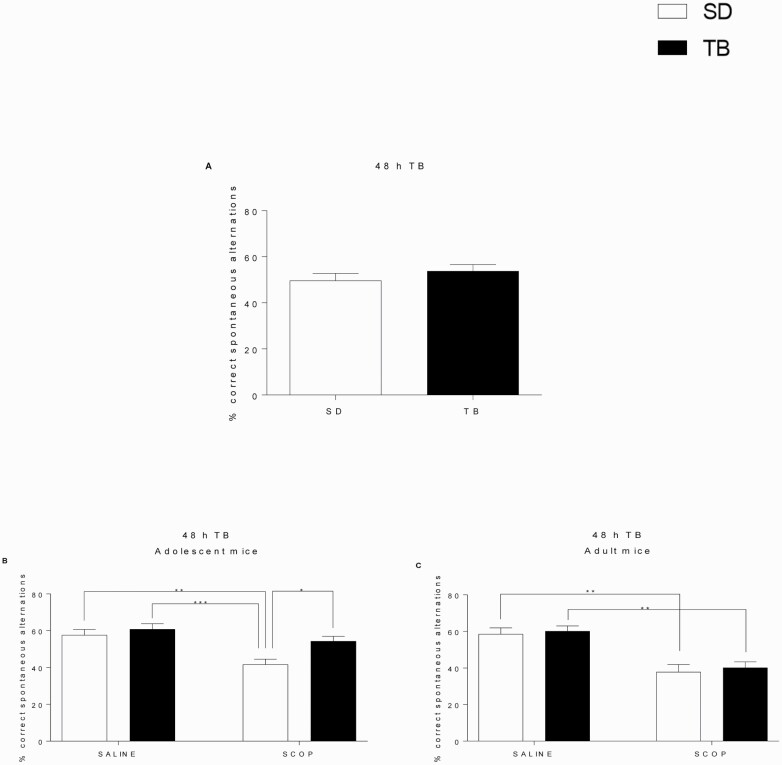
TB prevents SCOP-induced spatial memory deficits. (A) After 48-hour TB treatment, no differences between groups were observed in the percentage of spontaneous alternations (SD n = 6, TB n = 7). (B) TB prevented memory impairment caused by SCOP in adolescent mice (n = 8 per group). (C) In adult mice, SCOP-induced impairment was not prevented by TB (n = 8 per group). Data are expressed as means ± SEM. *P < .05; **P < .01; ***P < .001. Comparison was made by Student’s *t* test or 2-way ANOVA followed by post-hoc Bonferroni’s test.

The effect of TB on spatial working memory was initially characterized in adolescent mice that consumed the 1% TB diet (Assay 1). Figure 6A showed that the percentage of correct alternations in the Y maze was not altered by TB despite the effect of TB on LTP impairment evoked by SCOP, so a further experiment was designed to evaluate the capacity of TB to prevent the negative effect of SCOP on memory. The study was carried out in adolescent (5 weeks old) and adult mice (11 weeks old) (Assay 2). As illustrated in Figure 6, SCOP reduced the percentage of correct spontaneous alternations in both adolescent (Figure 6B) and adult (Figure 6C) animals. Nevertheless, the effect of SCOP was only prevented by TB in adolescent mice (Figure 6B; 2-ANOVA, SCOP effect F_(1,28)_ = 15.2, *P < *.001; TB effect F_(1,28)_ = 7.5, *P < *.05).

### TB Upregulated Gene Expression but Not Protein Levels of Glutamate Receptor Subunits Specifically in the Hippocampus

**Figure 7.**
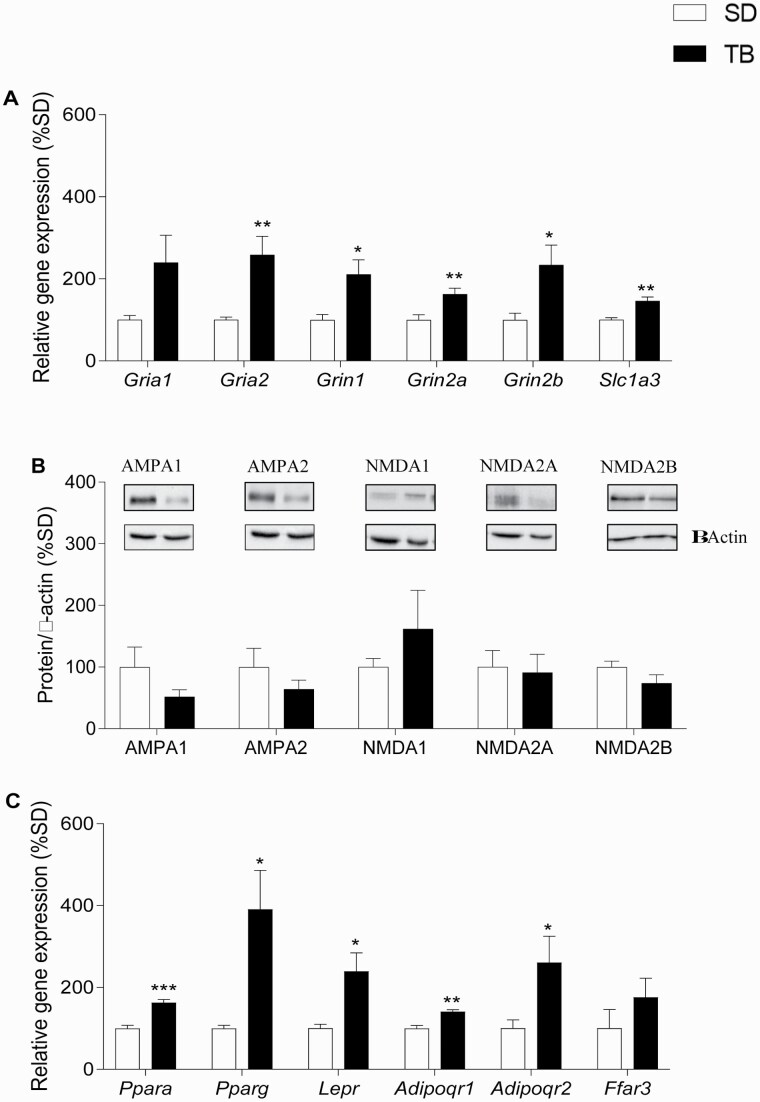
Effect of 48-hour TB diet on mRNA and protein expression levels of NMDA and AMPA receptors. (A) TB upregulated the expression of *Gria2*, *Grin1*, *Grin2A*, *Grin2b*, and *Slc1a3.* (B) Protein levels of AMPA receptor subunits 1/2 and NMDA receptor subunits 1/2A/2B were not modified by TB. (C) TB upregulated the expression of *Ppara*, *Pparg*, *Lepr*, *Adipoqr1*, *Adipoqr2*, and *Ffar3* genes within the HIP. Values are means ± SEM. (SD n = 6, TB n = 7). *P < .05; **P < .01; ***P < .001 compared with their respective controls (2-tailed Student’s *t* test).

Figure 7A shows the effect of TB on mRNA levels of genes encoding AMPA1/2 (*Gria1/2*), NMDA 1/2A/2B (*Grin1/2A/2B*) GLU receptor subunits, and the GLU transporter GLAST (*Slc1a3*). Statistical analysis revealed a significant effect of TB in the case of *Gria2* (*t*_(11)_ = 3.184, *P < *.01), *Grin1* (*t*_(11)_ = 2.772, *P < *.05), *Grin2A* (*t*_(11)_ = 3.126, *P < *.01), *Grin2B* (*t*_(11)_ = 2.444, *P < *.05), and *Slc1a3* (*t*_(10)_ = 4.217, *P < *.01). The effect was observed in the HIP but not in the PFC (Table 4), which indicates that TB has a selective impact within the HIP.

**Table 3.** BW and FI in Adolescent and Adult Mice.

	BW (g)		FI (kcal)	
	SD	TB	SD	TB
Adolescent mice	19.5 ± 0.4	19.4 ± 0.4	7.2 ± 0.5	6.8 ± 0.4
Adult mice	28.1 ± 0.7	26.6 ± 0.5	9.3 ± 0.9	8.3 ± 0.8

BW and FI was monitored during the 48-hour dietary treatment before Y Maze Test. Values are: Means ± S.E.M. (adolescent: SD n = 22, TB n = 23; adult: SD n = 16, TB n = 16) (adolescent: SD n = 6, TB n = 7).

**Table 4.** mRNA Levels in the prefrontal cortex after 48-hour TB Treatment

	Prefrontal cortex	
GENE	SD	TB
*Gria1*	100.0 ± 13.1	75.7 ± 7.8
*Gria2*	100.0 ± 20.6	84.6 ± 22.7
*Grin1*	100.0 ± 15.5	83 ± 26.4
*Grin2A*	100.0 ± 18.3	94.7 ± 14.4
*Grin2B*	100.0 ± 19.3	59.6 ± 20
*Slc1a3*	100.0 ± 17.9	70.3 ± 9.9
*Ppara*	100.0 ± 12.7	97.2 ± 15.4
*Pparg*	100.0 ± 28.2	65.7 ± 25.3
*Lepr*	100.0 ± 21.9	76.9 ± 22.9
*Adipor1*	100.0 ± 16.4	88.2 ± 26.2
*Adipor2*	100.0 ± 21.9	56.3 ± 15.6
*Ffar3*	100.0 ± 29.0	42.6 ± 9.8

Values are Means ± S.E.M. *Gria1* (Glutamate Ionotropic Receptor AMPA Type Subunit 1); *Gria2* (Glutamate Ionotropic Receptor AMPA Type Subunit 2); Grin1 (Glutamate Ionotropic Receptor NMDA Type Subunit 1); *Grin2A* (Glutamate Ionotropic Receptor NMDA Type Subunit 2A); *Grin2B* (Glutamate Ionotropic Receptor NMDA Type Subunit 2B); *Slc1a3* (Solute Carrier Family 1 Member 3); *PPara* (Peroxisome Proliferator Activated Receptor Alpha); *PParg* (Peroxisome Proliferator Activated Receptor Gamma); *Lepr* (Leptin receptor); *Adipor1* (Adiponectin receptor 1); *Adipo2* (Adiponectin receptor 1); *Ffar3* (Free Fatty Acid Receptor 3).

Nevertheless, although gene expression was regulated by TB, WB analysis revealed that protein levels of AMPA1, AMPA2, NMDA1, NMDA 2A, and NMDA2B receptor subunits were not modified by 1% TB (Figure 7B).

### TB Induced Expression of PPARγ, Adiponectin, and Leptin Receptor Genes Specifically in the Hippocampus

Because of the apparent implication of PPARγ on the effect of TB (Li et al., 2019), *Pparg* expression levels were quantified. As detailed in Figure 7C, *Pparg* expression was enhanced by TB (*t*_(10)_ = 2.524, *P < *.05) in the HIP but had no effect within the PFC (Table 4).

In addition, as illustrated in Figure 7C, TB also upregulated *Ppara* (*t*_(11)_ = 5.579, *P < *.001), *Lepr* (*t*_(11)_ = 2.779, *P < *.05), *Adipoqr1* (*t*_(11)_ = 4.259, *P < *.01), and *Adipoqr2* (*t*_(11)_ = 2.213, *P < *.05) expression (Figure 7C), specifically in the HIP of adolescent mice. No effect was found in the PFC (Table 4). The influence of TB on the expression of the SCFA receptor, FFAR3, was also investigated, although no effect (*t*_(10)_ = 1.15, *P* = .3) was identified (Figure 7C).

### Effect of TB on BW and Plasma Biochemistry

As appears summarized in supplementary Table 1, TB treatment had no effect on plasma biochemistry in adolescent mice. Moreover, neither BW nor FI were altered by the treatment (Table 3) in adolescent and adult mice.

## Discussion

Our results show that both TB and BA transformed e-LTP, generated by a single HFS train, into l-LTP, which usually requires the application of repeated HFS trains and engages transcription factors regulating de novo protein synthesis (Raymond and Redman, 2006). Therefore, these data allow us to speculate that TB/BA directly recruit proteins necessary to maintain LTP. Because transition from e-LTP to l-LTP involves the translation of mRNAs stored in synaptodendritic compartments (Pastalkova et al., 2006), an effect of TB/BA on the translation of pre-existing mRNA could be envisaged. Such a phenomenon, which underlies the so-called synaptic tagging (Frey and Morris, 1997), appears to concern a multiplicity of kinases, including calmodulin-dependent kinase II (Murakoshi et al., 2017), PKA (Huang and Kandel, 1994; Nguyen and Kandel, 1996), PKMζ, which is the constitutively active splice of PKC (Sacktor and Fenton, 2018), and the cellular energy and nutrient status sensor, AMPK (Marinangeli et al., 2018). Our study shows that AMPK inhibition with C-C blocked the effect of TB, suggesting that AMPK is recruited by TB to transform e-LTP into l-LTP.

The involvement of AMPK on synaptic plasticity appears to be controversial since some studies have evidenced that pharmacological activation of AMPK damps l-LTP maintenance (Potter et al., 2010) and also that AMPK inhibition alleviates the impairment of HIP synaptic plasticity induced by βA (Ma et al., 2014). Similarly, other researchers have reported an overactivation of neuronal AMPK in the brain of patients suffering from neurological disorders (Ju et al., 2011; Vingtdeux et al., 2011; Jiang et al., 2013). Nevertheless, the intra-hippocampus injection of adenovirus expressing AMPK has been shown to protect against SCOP-induced memory impairment (Kim et al., 2013), supporting the concept that AMPK activation is beneficial for learning and memory (Yu et al., 2016). In this vein, our current results suggest that the activation of AMPK by TB would account, at least partially, for the effect of this drug on LTP as well as on memory and synaptic plasticity. Moreover, our study is coherent with the general consensus regarding the beneficial impact that AMPK activation has on the neuroenergetic adaptation to LTP (Didier et al., 2018; Finley, 2018; Marinangeli et al., 2018). It must be highlighted that the in vitro effect of TB on AMPK activation and l-LTP formation could be unrelated with the improvement of memory observed in animals that consumed TB for 48 hours; in fact, it may appear striking that the incubation of HIP slices with TB during only 40 minutes is able to have an effect on HIP LTP. Nevertheless, our results are consistent with previous findings by other groups showing the enhancement of hippocampal LTP by BA under experimental conditions like that used in the current study (Levenson et al., 2004).

The ability of TB to rescue LTP in HIP slices that were perfused with SCOP indirectly supports the involvement of AMPK on TB effects, as muscarinic receptor activation has been shown to enhance AMPK activity in vitro (Thornton et al., 2008); therefore, one could expect that the effect of SCOP on LTP would be, at least partially, linked to the reduction of AMPK activity. Notably, the positive effect of TB on SCOP-induced impairment of memory is consistent with the study by Kim et al. (2013) showing that genetically engineered mice overexpressing AMPK within the HIP are protected against SCOP-induced memory impairment, a finding that is also coherent with the beneficial effect of AICAR (a pharmacological activator of AMPK) on cognition.

In regard to the mechanism that could account for AMPK activation, our study suggests the involvement of both LepR and AdipoQR2, which are known to regulate AMPK activity and whose corresponding mRNAs were increased by TB, specifically in the HIP. This regulation leads us to speculate that signaling pathways downstream of these receptors could account somehow for TB effects. This issue remains to be properly investigated, but it is noteworthy that both LepR and AdipoQR have been shown to be involved in HIP LTP (Irving and Harvey, 2014; Wang et al., 2019). Moreover, adiponectin and leptin play an important role in dendritic spine remodeling, neurogenesis, and synaptic plasticity in the HIP (O’Malley et al., 2007; Zhang et al., 2016).

Another relevant finding of this study deals with the blockade of TB effects by the PPARγ antagonist GW9662, which is coherent with the upregulation of *Pparg* gene expression and suggests that TB recruits PPARγ to modulate LTP. As a matter of fact, PPARγ is a target for SCFA (Li et al., 2014; Marion-Letellier et al., 2016), and therefore the involvement of PPARγ on TB effects would be expected. Notably, the role of PPARγ in cognitive and LTP performance has been identified in experimental models of neuronal seipin deficiency and aging (Zhou et al., 2016; d’Angelo et al., 2019), and PPARγ agonists have been reported to attenuate the βA peptide-dependent impairment of LTP (Costello et al., 2005). Taken together, the preceding findings reveal that transformation of e-LTP into l-LTP involves both AMPK and PPARγ and that these proteins are integral to the signaling pathway that accounts for TB effects. Interestingly, PPARγ activation has been shown to promote further activation and phosphorylation of AMPK (Lee and Kim, 2010).

Regardless of the role that AMPK and PPARγ may have on TB/BA effects, the increase of mRNAs encoding NMDAR and AMPAR subunits also suggests that adaptive mechanisms involving glutamate receptors participate in TB/BA-mediated responses. Nevertheless, mRNA and protein levels were apparently discrepant, since the increase of *Grin1*, *Grin2A*, and *Grin2B* mRNA was not associated with a similar upregulation of the NMDA1, NMDA2A, and NMDA2B subunits of NMDAR, which would be expected from the positive effect of TB on LTP. More surprisingly, in the case of *Gria1* and *Gria2* genes, their correlative proteins AMPA-1 and 2 tended to be reduced in TB-treated mice, despite the increase of the corresponding mRNAs. These findings do not necessarily imply that the density of functional NMDAR and AMPAR remains unchanged, since an influence of TB/BA on glutamate receptor subunit trafficking, leading to a different pattern of cytosolic/membrane protein distribution, cannot be discarded (Penn et al., 2017). Otherwise, the absence of correlation between mRNA and protein content could be due to spatial and temporal variations of local availability of resources because protein biosynthesis/degradation and gene transcription are, in many cases, unmatched (Liu et al., 2016).

With regard to the effect of TB on HIP-dependent memory, our findings show that this drug reverses memory impairment evoked by SCOP in the Y maze. This result is coherent, with some studies indicating that BA enhances LTP and memory (Levenson et al., 2004; Kim et al., 2013; Zhong et al. 2014; Silva et al., 2020). Notably, BA has been shown to protect and reverse learning and memory deficits associated with AD (Fernando et al., 2020) as well as HFD-induced cognitive impairment (Arnoldussen et al. 2017). It is important to note that the protective effect of TB on SCOP-induced impairment of spatial memory was not observed in adult mice, which is a limitation for the eventual use of TB in neurodegenerative diseases. Therefore, further studies in aged mice or in mice models of neurodegenerative diseases should be carried out to further characterize the effect of TB. In any case, our results are in accordance with other research showing that the beneficial effects of dietary fiber, which is a main source of BA, are specifically observed in adolescent mice (Track et al., 1985).

Finally, we have identified the expression of FFAR3 within the HIP, which suggests that TB/BA effects could be mediated by this receptor, which is one of the 2 G-protein coupled receptors (FFAR2 and FFAR3, formerly GPR43 and GPR41) (Le Poul et al., 2003; Tang et al., 2015) able to bind SCFA in the brain. This finding led to speculate that FFAR3 might be a potential target for memory enhancers useful to delay cognitive decline.

In summary, our study shows that TB elicits favorable changes, both in HIP synaptic plasticity mechanisms as LTP and spatial memory, which suggests that BA may be a promising tool endowed with therapeutic potential in memory impairment conditions.

## Supplementary Material

pyac015_suppl_Supplementary_Table

pyac015_suppl_Supplementary_Figure

## Abbreviations

AD: Alzheimer disease
AMPK: AMP-activated protein kinase
AP-5: L-2-amino-5-phosphonovaleric acid
βΑ: β-amyloid
BA: Butyric acid
BW: Body weight
BST: Basal synaptic transmission
CA: *Cornu Ammonis*
C-C: C-Compound
DMSO: Dimethyl sulfoxide
EIA: Enzyme immunoassay
e-LTP: Early LTP
fEPSP: Field excitatory post-synaptic potentiation
FI: Food intake
**GLU**: Glutamate
HDAC: Histone deacetylase
HFD: High-fat diet
HFS: High frequency stimulation
HIP: Hippocampus
I/O: Input/Output
KRB: Krebs ringer bicarbonate
LTP: Long-term potentiation
l-LTP: Late LTP
NEFA: Non-esterified free fatty acids
PFC: Prefrontal cortex
SCFA: Short chain fatty acids
SCOP: Scopolamine
SD: Standard diet
SEM: Standard error mean
TB: Tributyrin
