# Supplementary material for: Butyric Acid Precursor Tributyrin Modulates Hippocampal Synaptic Plasticity and Prevents Spatial Memory Deficits: Role of PPARγ and AMPK
Source: Int J Neuropsychopharmacol. 2022 Feb 13;25(6):498–511. doi: 10.1093/ijnp/pyac015 (PMC9211015; doi:10.1093/ijnp/pyac015)
Supplement: pyac015_suppl_Supplementary_Table [file pyac015_suppl_supplementary_table.docx]

**Supplementary Material**

**Supplementary Table S1. Effect of 48-h treatment with TB on plasma parameters in adolescent mice**. Values are means ± S.E.M. (SD n=6, TB n=7).

|  | **SD** | **TB** |
| --- | --- | --- |
| **Glucose (mg/dL)** | 167.9 ± 24.7 | 166.6 ± 11.9 |
| **Triglycerides (mg/dL)** | 167.2 ± 30.5 | 168 ± 17.1 |
| **NEFA (mg/dL)** | 65.5 ± 8.4 | 69.3 ± 6.9 |
| **Adiponectin (µg/mL)** | 2.5 ± 0. 4 | 2.2 ± 0.1 |
| **Insulin (ng/mL)** | 0.90 ± 0.17 | 0.70 ± 0.30 |
| **Leptin (ng/mL)** | 2.6 ± 0.2 | 2.9 ± 0.3 |

**Supplementary Figure S1. Complete set of immunoblots** of proteins AMPA1/2, NMDA1/2A and NMDA2B with their respective β-actin. In all the gels, the order of the samples on the membrane is the same: 1% TB, 3% TB and SD up to a total of 8 samples per membrane. Data from 3% TB dose are not showed in the present manuscript.
