# Supplementary material for: Butyric Acid Precursor Tributyrin Modulates Hippocampal Synaptic Plasticity and Prevents Spatial Memory Deficits: Role of PPARγ and AMPK
Source: Int J Neuropsychopharmacol. 2022 Feb 13;25(6):498–511. doi: 10.1093/ijnp/pyac015 (PMC9211015; doi:10.1093/ijnp/pyac015)
Supplement: pyac015_suppl_Supplementary_Figure [file pyac015_suppl_supplementary_figure.pptx]

## Slide 1
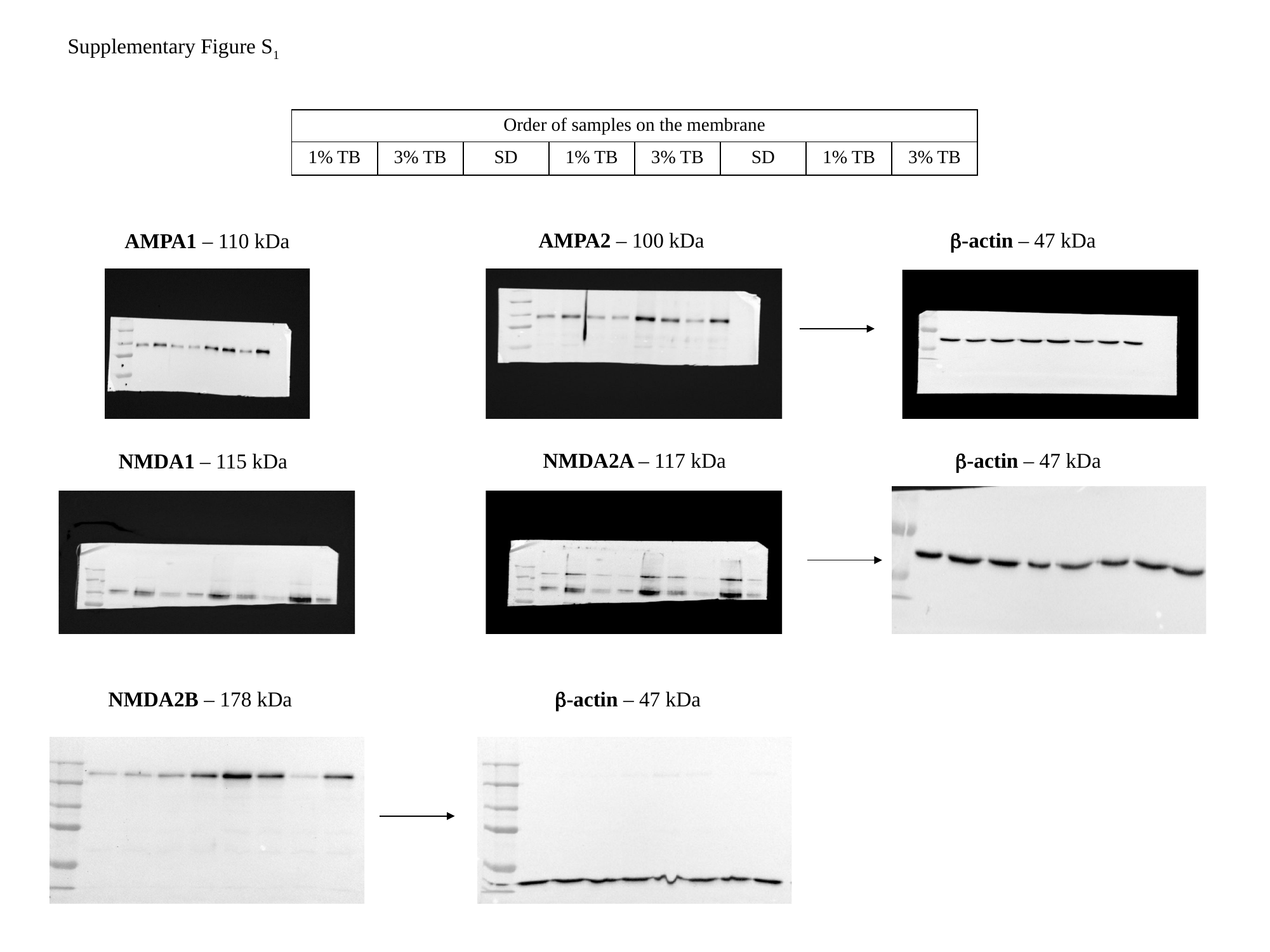

Supplementary Figure S1
| Order of samples on the membrane | | | | | | | |
| --- | --- | --- | --- | --- | --- | --- | --- |
| 1% TB | 3% TB | SD | 1% TB | 3% TB | SD | 1% TB | 3% TB |
b-actin – 47 kDa
AMPA2 – 100 kDa
AMPA1 – 110 kDa
NMDA2A – 117 kDa
b-actin – 47 kDa
NMDA1 – 115 kDa
NMDA2B – 178 kDa
b-actin – 47 kDa
